# Clinical implication of genetic composition and molecular mechanism on treatment strategies of HER2-positive breast cancers

**DOI:** 10.3389/fonc.2022.964824

**Published:** 2022-10-31

**Authors:** Christopher Y.C. Chow, Erich Ferdiansyah Lie, Cheng-Hsun Wu, Louis W.C. Chow

**Affiliations:** ^1^ UNIMED Medical Institute, Hong Kong, Hong Kong SAR, China; ^2^ Graduate Institute of Biomedical Sciences, China Medical University, Taichung, Taiwan; ^3^ Department of Anatomy, China Medical University, Taichung, Taiwan; ^4^ Organisation for Oncology and Translational Research, Hong Kong, Hong Kong SAR, China

**Keywords:** genetic, molecular, HER2, breast cancer, treatment, strategy

## Abstract

The current clinical management model of HER2-positive breast cancers is commonly based on guidelines, which in turn are based on the design and outcome of clinical trials. While this model is useful to most practicing clinicians, the treatment outcome of individual patient is not certain at the start of treatment. As the understanding of the translational research of carcinogenesis and the related changes in cancer genetics and tumor microenvironment during treatment is critical in the selection of right choice of treatment to maximize the successful clinical outcome for the patient, this review article intends to discuss the latest developments in the genetic and molecular mechanisms of cancer progression and treatment resistance, and how they influence the planning of the treatment strategies of HER2-positive breast cancers.

## Introduction

Breast cancer is a common health problem. The GLOBOCAN 2020 estimates of cancer incidence and mortality released by International Agency for Research on Cancer in 2021 showed that female breast cancer has surpassed lung cancers as the leading cause of global cancer incidence with an estimate of 2.3 million new cases and accounting for 11.7% of all cancer cases (1). The incidence rates are rising fast in South America, Africa, and Asia, particularly in high-income Asian countries such as Japan and Korea.

Of the different subtypes of breast cancer, human epidermal growth factor receptor-2 (HER2)-positive cancers remain a significant health care burden despite advances and improvements in treatment (2). HER2 enriched cancers account for approximately 20% of all breast cancers and are associated with a higher relapse rate and a significantly shorter survival (3).

The current clinical management model of these cancers is commonly based on guidelines, which in turn are based on the design and outcome of clinical trials. While this model is useful to most practicing clinicians, the treatment outcome of individual patient is not certain at the start of treatment. Precision medicine and oncology treatment emphasize the importance of treatment basing on the specific cancer characteristics of individual patient. Moreover, over the past decade, the armamentarium of treatment choices has increased substantially for HER2-positive breast cancer. Thus, the understanding of the translational research of carcinogenesis and the related changes in cancer genetics and tumor microenvironment during treatment is critical in the selection of right choice of treatment to maximize the successful clinical outcome for the patient.

## The signal transduction pathways in HER2-positive cancers

HER2 is one of the four members of the HER family, which is localized to chromosome 17q and is a tyrosine kinase receptor protein that encodes cell to cell communication *via* signaling transduction. The cellular activities start with the dimerization of the HER2 receptors (4). HER2 can dimerize with itself or other HER family members and the resultant phosphorylated dimer activates the tyrosine kinase domain (TKD) of the intracellular component of the receptor. The activated TKD can in turn recruit different signal transduction pathways in the downstream cascades leading to cell survival and proliferation, as well as resistance to treatment.

Among the downstream signaling pathways, the PI3K-AKT pathway, the NF-kappaB pathway, and the MAPK pathway are more frequently reported in HER2-positive breast cancer. However, it seems that during the early cellular events, the PI3K and the NF-kappaB pathways are more important.

### Phosphatidylinositol-3-kinase and the effector protein B pathway

Amplification of HER2 *via* homodimerization and heterodimerization drives tumorigenesis. As HER3 has six consensus binding sites for the recruitment of the Src homology 2 (SH2) domain of PI3K, it is regarded as the potent activator of PI3K (5). However, subsequent experiments show that HER2 can directly bind to PI3K especially at high expression and phosphorylation levels (6). The progressive functional gains by HER2 can activate PI3K and overcome its dependency on HER3.

**Figure 1:**
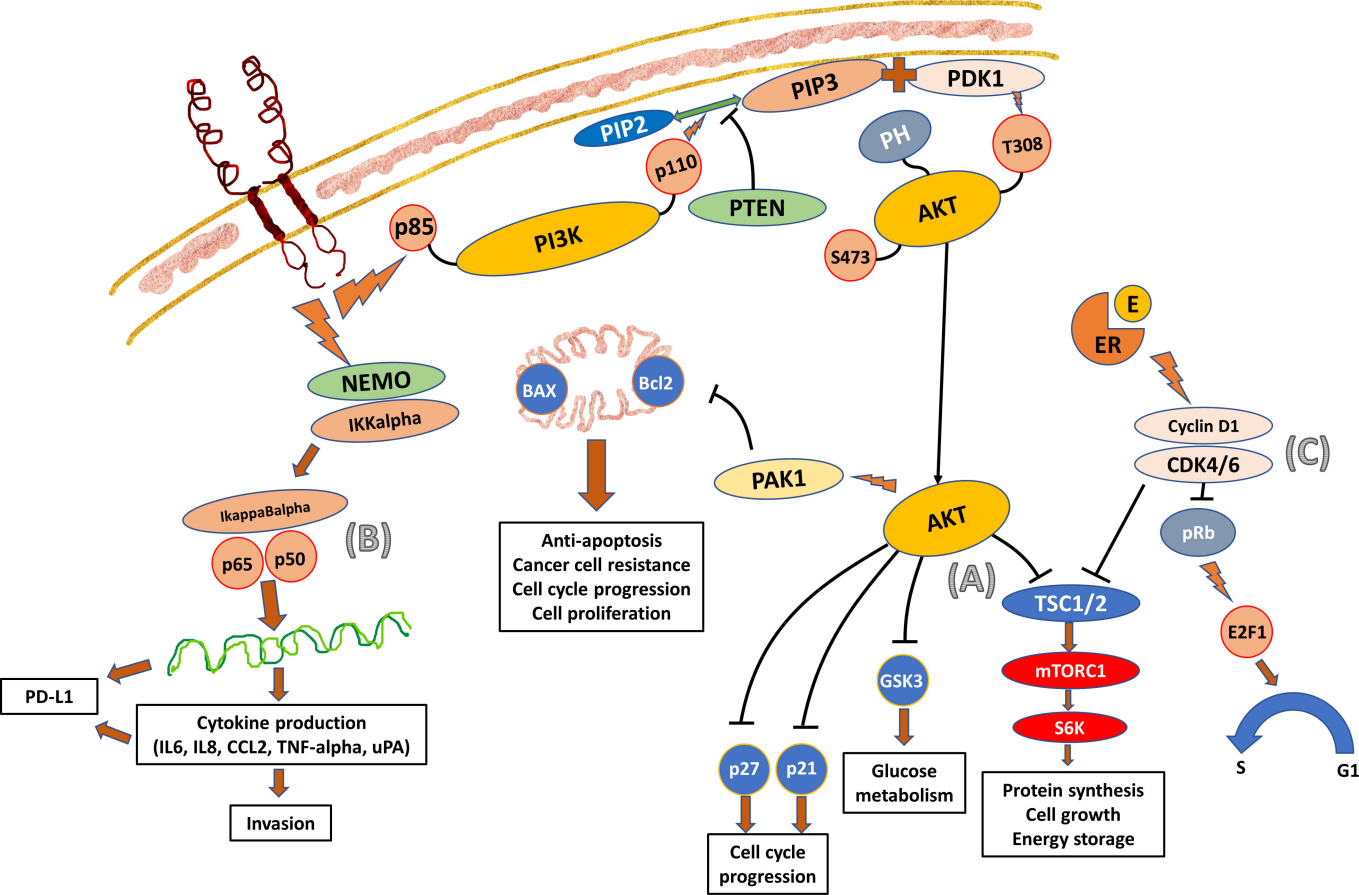
Crosstalk Between the PI3K/AKT, NF-kappaB, and Cyclin D1-CDK4/6-pRb Pathways. **(A)** PI3K is a heterodimeric protein consisting of regulatory (p85) and catalytic (p110) components. The p85 regulatory subunit contains two SH2 groups. PI3K interacts with Ptdlns (3–5) P3 (PIP3) and the effector AKT. The activated TKD of HER2 receptor recruits PI3K towards the cell membrane and activates the p85 regulatory components (7). The p110 catalytic component then in turn phosphorylates the PIP3 membrane phospholipid which recruits the plekstrin homology (PH) domain of the AKT protein. The activated PIP3 also binds to 3-phosphoinositol-dependent protein kinase-1 (PDK1) allowing it to access and phosphorylate the Thr308 catalytic unit of AKT. The activated AKT then moves to the cell to activate a number of cellular processes stimulating cell growth and inhibiting cell apoptosis. One of the main roles is to phosphorylate and inhibit tuberous sclerosis complex (TSC) subunits 1 and 2 (TSC1/2) and activate mTOR complex 1 (mTORC1), which in turn activates ribosomal protein S6 kinase (S6K) and ribosomal S6 to promote protein synthesis and cell growth (8). **(B)** NF-kappaB can be activated by the classical canonical cascade starting from the cell surface receptor and by the alternative non-canonical cascade. The signal from the activated TKD of HER2 receptor is transferred into the cytoplasm and activates NF-kappaB by the classical canonical pathway (9). The activation involves IKKalpha and IKKbeta catalytic subunits of the IkappaB kinase (IKK) complex which degrades the IkappaB inhibitory protein of NF-kappaB. As a result, the released NF-kappaB dimers translocate to the nucleus and activate the downstream gene transcription, producing cytokines (IL-6, TNF-alpha, IL-4 and IL-5), chemokines (IL-8, RANTES, MIP-1alpha, MCP-1), adhesion molecules, and other enzymes that lead to the influx of T-cells, B-cells, monocytes, dendritic cells, and NK-cells into the tumor. The dynamics of these tumor infiltrating lymphocytes (TIL) will determine the aggressiveness and invasiveness of the cancer. It is well documented that cancers with higher percentage of TIL have a better response to treatment and survival outcome. **(C)** CDK4/6 interacts with cyclin D1 and inactivates the retinoblastoma (Rb) tumor suppressor protein (pRb) to promote the transcription factor E2F1 to control cell cycle transition from G1 to S phase (10). The cyclinD1-CDK4/6 complex is also responsible for the phosphorylation of TSC 1 and 2, which in turn leads to the phosphorylation of S6K and mTORC1, one of the downstream signal cascades of AKT.

As shown in **Figure 1A**, the activated PI3K can be deactivated by phosphatase and tensin homolog (PTEN), a tumor suppressor gene that encodes a phosphatase to switch PIP3 to Ptdlns (4, 5) P2 (PIP2). The switch inhibits the downstream signal transduction of PI3K and thus suppresses cell proliferation and enhances apoptosis (11). However, the downstream signals of PI3K/AKT rather than the inhibitory switch predominate in cancer cells, especially when there are mutation and deletion of PTEN gene leading to PTEN loss (12, 13). The resultant hyperactivation of PI3K pathway promotes tumorigenesis and metastasis. PTEN mutation or loss occurs less frequently at diagnosis but is associated with disease progression (14, 15).

### Mitogen-activated protein kinase

MAPKs are important mediators of intracellular signaling in tumorigenesis. The MAPK pathway has been implicated as one of the most important pathways of cancer growth and metastasis in many types of cancers. It consists of three major signaling pathways, including the extracellular signal-regulated kinase (ERK) pathway, the c-Jun N-terminal kinase (JNK) pathway, and the p38 pathway (16). Each of the three pathways has a three-tiered cascade of protein kinases, which form an extensive MAPK toolkit when they combine with other functional components such as receptors and transducers. The signal is usually rapidly carried downstream to different targets of biological functions resulting in cell proliferation (ERK and JUN pathways), apoptosis (JUN and p38 pathways), and cytokine release (p38 pathway).

Of the two pathways described, notably PI3K/AKT and MAPK pathways, it has always been the former that is frequently linked with HER2-positive breast cancer, especially during the early stages of the cancers, whereas MAPK activation and its activities occur more often in the metastatic setting or in cancers resistant to treatment (17). A study that investigated the impact of genetic and pharmacologic manipulation of the PTEN pathway in a ribonucleic acid (RNA) interference mouse model of HER2 metastatic breast cancer shows that the disease progression from PTEN silencing is related to elevated signaling through PI3K and MAPK (MEK) pathways (18).

### NF-kappaB pathway

NF-kappaB is a family of transcription factors that plays an important role in innate and adaptive immune response. Its dysregulated function may also be involved in cancer development and the activation is commonly observed in cancer. It was reported that activated NF-kappaB was more often detected in estrogen receptor (ER)-negative cancers than ER-positive cancers, and mostly in ER-negative and HER2-positive breast cancers (86%) (19). Among the ER-negative breast cancers, the active NF-kappaB-DNA binding activity was elevated in HER2-positive cancer cells. Inhibition of the NF-kappaB pathway and its activation decreased cell proliferation and increased apoptosis (**Figure 1B**).

It has been suggested that the NF-kappaB pathway can be activated by the downstream cascade of PI3K/AKT (20, 21). However, experiments using PI3K inhibitor indicated that NF-kappaB is not activated by PI3K in HER2-positive cells (9). This inhibition reduced the cell proliferation, but not cell invasiveness. The study further showed that knockdown of IKKalpha, the subunit that is usually involved in the non-canonical pathway but found to be involved also in the canonical pathway in HER2-positive cells, resulted in a decrease in cell invasion. Thus, HER2 signal transduction utilizes both PI3K and NF-kappaB pathways independently to promote cell growth and invasion.

### Cyclin-dependent protein kinase pathway

The importance of CDK has been extensively studies in hormone receptor-positive breast cancers. CDKs are involved in cell cycle transition, and CDK4/6 in particular regulates cell proliferation (**Figure 1C**).

The cross talk between the cyclinD1-CDK4/6-pRb axis and PI3K/AKT/mTOR pathway has always been regarded as more obvious in hormonal receptor-positive and HER2-positive cancers, as ER can directly target cyclin D1 transcriptionally and activate CDK4/6-pRb. However, when the levels of pRb, as a reflection of CDK4/6 activity, were evaluated with respect to HER2 status, it was found that ER-negative and HER2-positive cancers had the highest level. This was followed by ER-positive and HER2-positive cancers and then HER2-negative cancers with the lowest level of pRb (22). Thus, cross talk exists between the two pathways irrespective of the hormonal receptor status.

### Immune checkpoints pathway

Immune checkpoint factors, programmed cell death protein-1 (PD-1) and its ligand programmed cell death-ligand 1 (PD-L1), have been subjects of intense cancer research. PD-1 is a transmembrane receptor commonly found on the surface of T-cell, NK cell, B-cell, dendritic cells, monocytes, and macrophages (23). PD-1 and cytotoxic T-lymphocyte-associated protein 4 (CTLA-4) are involved in the regulation of the innate and adaptive immune response. They provide the ‘braking’ mechanism to halt the immune function to prevent excessive inflammatory response (24). PD-L1 is another transmembrane protein usually expressed by T-cell, B-cell, dendritic cells, and cancer cells. The binding of PD-L1 to PD-1 promotes immune evasion in the tumor microenvironment leading to a reduction in cytokine secretion as well as inhibition of T-cell activation, proliferation, and survival.

PD-1 and PD-L1 are growing to be important targets to treat triple negative breast cancer by immunotherapy. However, they may also play a role in HER2-positive breast cancers. PD-L1 expression has been detected in 48.5% of primary HER2-positive cancers and 30.5% of the associated TILs (25). Among the PD-L1-positive cancers, 53.1% also showed high expression of PD-L1 in TILs. In another study evaluating the clinicopathological values of PD-L1 expression in HER2-positive breast cancer, the PD-L1 positivity rate was 17.5% and there was also a significant correlation of positivity with high degree of TILs (26). Furthermore, the pathologic response (pCR) rates after neoadjuvant chemotherapy in combination with trastuzumab were related to PD-L1 expression and TILs.

**Figure 2:**
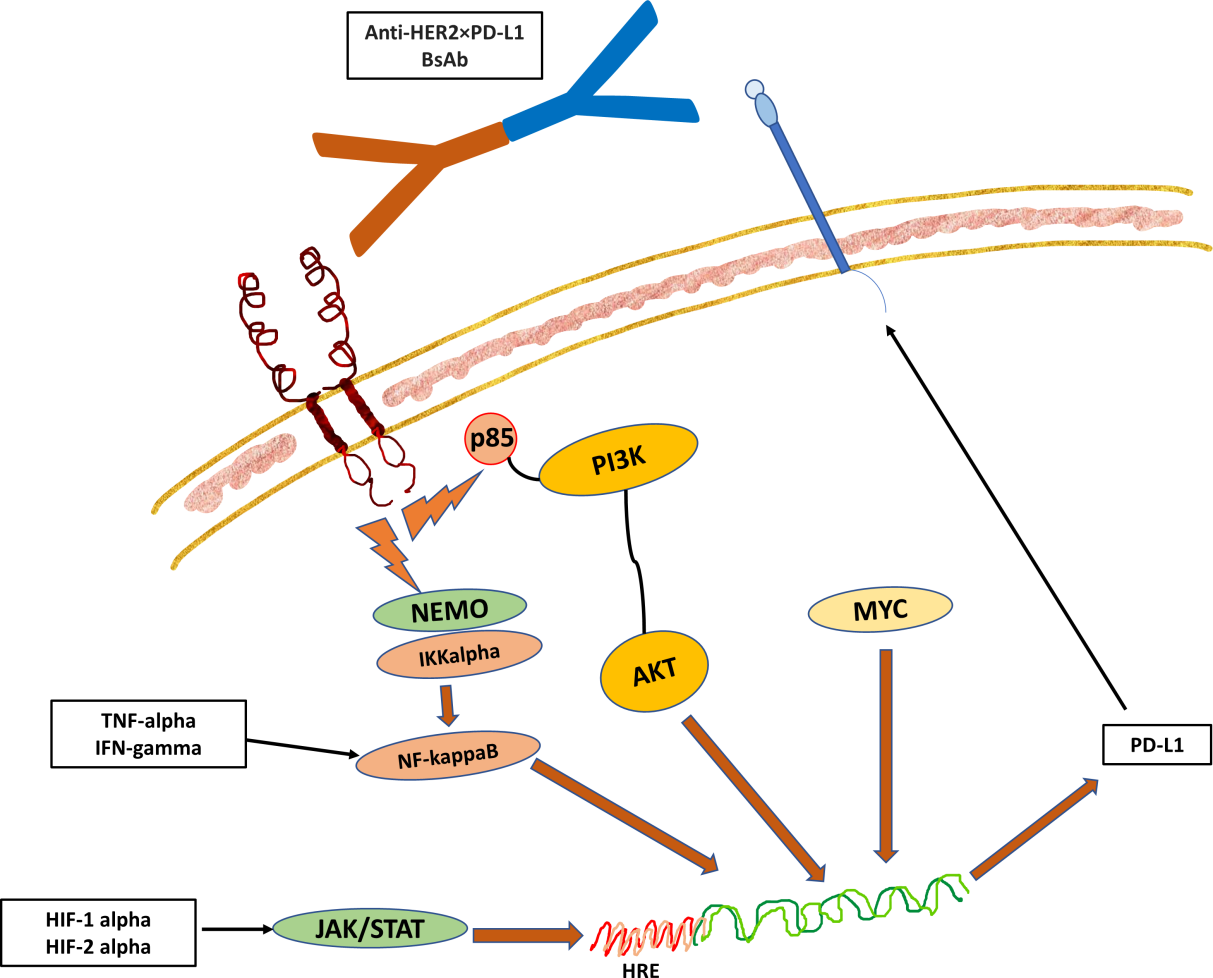
The Hypothesized Role of NF-kappaB Pathway in the Regulation of the Immune Checkpoint Pathway. The downstream signal of receptor activation triggers the translocation of NF-kappaB into the nucleus, which then binds to the promoter region of the PD-L1 gene (27). In addition, the changes in the dynamics of the tumor microenvironment such as hypoxia and cell stress with surge of pro-inflammatory cytokines such as TNF-alpha and IFN-gamma can also drive the NF-kappaB pathway to induce the expression of PD-L1 in cancer cells or TILs. While there is an association of HER2 expression with PD-1/PD-L1 pathway, the exact mechanisms that link the two together remain to be elucidated. However, it is likely that PD-L1 expression indicates epithelial-mesenchymal transition (EMT) in cancers to protect mesenchymal cells and stem cells from immune attack. This hypothesized crosslink activity between HER and PD-L1 forms the basis of development of BsAb targeting both HER2 and PD-L1.

There are several signal transduction pathways that influence the expression of PD-L1, which include the P13K-AKT pathway, the MAPK pathway, the JAK-STAT pathway, the WNT pathway, the Hedgehog (Hh) pathway, and the NF-kappaB pathway. Among the signal transduction pathways that are linked to PD-1/PD-L1 pathway, NF-kappaB seems to play a key role in the regulation of this immune checkpoint pathway in cancers (**Figure 2**). Besides these signal transduction pathways, the PD-1/PD-L1 pathway can also be influenced by microRNAs (miRNAs) and long non-coding RNA (lncRNAs). The miRNAs are small molecules that regulate gene expression through transcriptional regulation of mRNA. Examples that were found to enhance the expression of PD-L1 include miR-155 and miR-146a (28, 29). There are also miRNAs that inhibit the PD-L1 expression such as the miR-33a, miR-34, miR-21, and miR-873 (30–33). lncRNAs are involved in transcriptional editing and they are also found to be involved in the regulation of the immune response *via* enhancing or inhibiting PD-L1 expression. Examples include AFAP-1-AS1, UCA1, and SNHG20 that enhance PD-L1 expression, whereas NKX2-1-AS1 and MALAT1 inhibit PD-L1 expression (34–38).

## Factors of metastasis and drug resistance

### Mutations of HER2 receptor

Mutations or changes in HER2 amplification and its downstream pathways described above may affect the effectiveness of cancer therapies leading to treatment resistance and metastasis.

Monoclonal antibody treatment with trastuzumab or pertuzumab requires the binding of the antibodies with the extracellular domain (ECD) of the HER2 receptor. Such binding promotes natural killer (NK) cell activation and mediates the antibody-dependent cell-mediated cytotoxicity (ADCC) (39). Besides the ADCC activation, the binding also leads to inhibition of ECD cleavage, abrogation of intracellular signaling, reduction of angiogenesis, and decreased DNA repair (40). However, primary resistance, a lack of intrinsic response to treatment, is not uncommon. It has been described that over 60% of HER2-positive breast cancers develop primary resistance to single agent trastuzumab (40). Targeting both HER2 receptors with dual antibodies or with inhibitors of other pathways may enhance the benefit of trastuzumab and overcome potential primary resistance.

Trastuzumab resistance could arise from the HER2 receptors when they mutate or when the ECD is truncated. In a report studying the HER2 somatic mutations in human breast cancers, it was found that the prevalence was not high in primary tumors with an overall rate of 2.24% (2.31% for HER2-positive cancers and 2.07% for HER2-negative cancers) (41). However, the mutation rate was much higher in metastatic HER2-positive cancers at 27.8%. The most frequent mutation of primary HER2-positive cancers is K753E and that of HER2 negative cancers are L768S and V773L. But for metastatic HER2-positive cancers, L755S and K753E were identified and confirmed to be drug resistant mutations. These mutated receptors could autophosphorylate and activate the MAPK and JNK/SAPK pathways leading to trastuzumab and lapatinib resistance (42). Similar results were also reported that L755S mutation could confer hyperactivation of MAPK and PI3K/AKT pathways leading to resistance to lapatinib and neratinib (43). However, when these tyrosine kinase inhibitors (TKIs) were combined with MEK inhibitor or PI3K inhibitor, robust killing of cancer cells was observed. Besides these mutations, del.16 was another frequently reported mutation which could lead to the activation of SRC pathway and oncogenic transformation (44, 45). Furthermore, it was shown that cancer cells with HER2-del.16 and HER2-L755S were more responsive to TKI than cells with HER2-L755S alone.

When circulating tumor DNA (ctDNA) of the peripheral blood was analyzed, HER2 somatic mutations were detected in 8.9% of metastatic breast cancer patients and they were associated with shorter progression-free survival (PFS) (46). The HER2-positive cancers had a higher mutation frequency and variant allele frequency than HER2-negative cancers (19.5% vs. 4.8%). This may be related to HER2 gene copy number gain. In addition, HER2 mutations in HER2-positive cancers were more likely to occur in ECD (44.2% vs. 25.0%), whereas the mutations that occurred in HER2-negative cancers were more likely to occur in the kinase domain (25.8% vs. 57.7%). HER2-negative cancers with ECD mutations showed greater response to pyrotinib than those cancers with TKD mutations. Notably, 95.5% of HER2 mutations were associated with subclonal tumors; the value of the HER2-positive cancers was greater than HER2-negative cancers (96.9% vs. 82.7%). The most frequent co-occurring mutations included TP53, PIK3CA, MLL3, FAT2, NF1, and GATA3. It was postulated that these HER2 mutations might be induced by anti-cancer therapy and trastuzumab leading to drug resistance and metastasis (47). However, these cancers with induced mutations might still respond to TKI such as neratinib, though they showed trastuzumab resistance (48).

### HER2 gene copy number and HER2 heterogeneity

Cancers with a higher HER2 GCN as identified by FISH test (i.e >12) showed a higher response rate to trastuzumab than those with intermediate copy number (i.e. 6-12) (49, 50). GCN was also found to be prognostic in patients with HER2-positive metastatic breast cancer, as patients with six or more copies of HER2 showed a significantly better overall survival rate (51) However, it should be highlighted that high GCN was associated with higher chances of brain metastasis despite a more favorable extra-cranial response to treatment. Besides studying tissue samples, the use of liquid biopsy showed that high plasma HER2 GCN was prognostic in metastatic breast cancer (52). It could also be used to monitor treatment response to trastuzumab, as patients who were responsive to treatment had a significant decrease in HER2 GCN on serial measurement. It was recommended that HER2 GCN should be regarded as a biomarker of trastuzumab efficacy and prognosis.

Gene expression profiling studies on pre- and post-treatment samples of neoadjuvant anti-HER2 study CALGB-40601 showed that HER2 enriched cancers had a higher pCR rate than other types (70% for HER2 enriched vs. 36% for luminal B vs. 34% for luminal A) (53). Analysis of the post-treatment residual cancer samples showed that these non-pCR cancers could switch to other tumor types and mostly to luminal A. This switch was considered to be more related to tumor heterogeneity than from treatment. The KRISTINE study also showed that tumor heterogeneity could be a marker of pCR with lower HER2 mRNA expression showing a higher loco-regional progression while on treatment and homogenous staining occurred mostly in non-progressive patients (54). Similarly, intratumoral heterogeneity was also found to be associated with pCR with non-heterogenous subset showing a much higher pCR than HER2-heterogenous subset (55). The former subset could be considered for HER2 targeted therapies without chemotherapy, whereas addition of chemotherapy could be considered for the latter subset.

### Truncated HER2 receptor

Truncated HER2 receptor is also called p95, named after its molecular weight. The lack of the ECD domain contributed to trastuzumab resistance as the monoclonal antibody was not able to bind to the receptor and prevent the subsequent signal transduction. The truncated receptor could be a result of metalloproteases cleavage of the ECD or related to alternative initiation of mRNA translation (56, 57). The loss of ECD is not uncommon and p95 is associated with higher tumor load, locoregional, and distant metastasis and shorter disease-free survival (58, 59). In a study of 32 patients who developed metastasis from trastuzumab-based chemotherapy failure, p95 was expressed in 34.4% of patients who had more visceral metastasis and worse overall survival (60). Although p95 over-expressed cancers have a higher rate of trastuzumab failure, they could be responsive to TKI as their kinase activity is retained (61, 62). As p95 could be identified by immunohistochemistry, determination of p95 expression among HER2-positive cancers could be a simple but effective biomarker of trastuzumab efficacy.

### Gene mutational changes

Extensive translational studies and analysis were performed on the genetic changes leading to treatment resistance and metastasis. DNA gene sequencing studies on diagnostic samples showed high rate of mutations in over 70% of HER2-positive breast cancers (63). The most frequently mutated genes were TP53 and PIK3CA, which were very often involved in cancer progression. TP53 mutation was reported in over 50% of cancers, but the rate was higher in another study analyzing cancers in Chinese patients (74.6%) (64). TP53-mutated cancers were associated with a poorer survival outcome (65). This might be related to its induction of cell arrest and senescence instead of apoptosis as well as promoting production of cancer stem cells (66, 67). Analysis of the pre-treatment samples of patients receiving neoadjuvant anti-HER2 treatment showed that TP53 mutation was commonly found in the pCR subgroup than non-pCR subgroup (67% vs 43%) and TP53 LOF mutation (and initial Ki67) was predictive of pCR status (63, 64).

Mutational changes of the signal transduction pathway involving the PI3K/AKT pathway occurs in 20% to 40% of breast cancers and PI3KCA is a negative prognostic factor (68). The mutation in HER2-positive cancers may overcome trastuzumab blockage of the HER2 receptor dimerization and auto-activate its down-stream signal. Such mutation alone or when there was also PTEN loss was associated with a shorter disease-free survival and overall survival in the adjuvant anti-HER2 treatment trials (69, 70). Neoadjuvant trials on trastuzumab alone or in combination with lapatinib showed that PI3KCA mutation was identified more commonly in those patients who had residual cancers after treatment (53, 71–73). Higher pCR rate was found in PI3KCA mutated HER2-positive and hormonal receptor-positive cancers than hormonal receptor-negative cancers (68). Irrespective of hormonal receptor status, PI3KCA mutation rate of the primary cancers was similar in both pCR and non-pCR subgroups (27% vs. 28% respectively) in contrast to the earlier finding that PI3KCA and RhoA mutation were associated with higher pCR (63, 72). Moreover, it was not found to be a predictive factor of pCR (64). Thus, PI3KCA mutation could merely be the genetic changes related to cancer progression instead of involving in treatment resistance.

Besides switching from HER2 enriched to luminal A cancers, selective pressure from treatment could change the gene mutational profile. In more than half of residual cancers after neoadjuvant anti-HER2 treatment showed increase in total number of detected mutations when compared to the original primary tumor diagnostic samples and in a small percentage, the mutational profile was completely different (63). The mostly detected mutated genes were PI3KCA, TP53, MET, NOTCH, FGFR3, and PTEN. While the mutational changes in the primary cancers was not associated with prognosis, residual disease, especially those cancers with changes in gene mutational profile after neoadjuvant treatment, significantly influenced rate of relapse (42% with mutational changes relapsed).

Though PI3KCA mutation might not be a predictor of pCR in neoadjuvant anti-HER2 studies, a study evaluating the efficacy of buparlisib, a pan-PI3K inhibitor, showed that blockage of both PI3K and HER2 led to a higher objective response rate (ORR), implying that PI3K pathway could still be an important pathway affecting treatment efficacy and metastasis (74).

Survival analysis showed that the loss of PTEN in this PI3K pathway was also associated with a poorer survival outcome with shorter disease-free survival and overall survival (69). The blockage of HER2 receptor by both trastuzumab and pertuzumab might benefit high-risk patients with a better disease-free survival rate. DNA analysis showed that PI3K alteration was associated with a worse outcome and decreased the pertuzumab benefit (70). Similarly, PI3KCA mutation was an important but negative predictive marker of pertuzumab efficacy in metastatic breast cancers (75). Patients with PI3KCA mutation had a shorter medial PFS though treated with dual targeted therapies.

PI3KCA mutation is a marker of the innate aggressive nature of cancers and it could be related to PTEN loss and increase in PI3K activation. It is also possible that treatment may provide additional selective pressure for more mutational changes. As a result, the cancers become even more aggressive and metastasize. In a study evaluating PTEN suppression and PI3K activation, it was observed that MAPK pathway was activated as well (18). Inhibition of MAPK signaling and PTEN restoration triggered similar tumor regression. In another study evaluating the inhibition of PI3K pathway, it was reported that the inhibition could activate compensatory pathway of ERK signaling (76). The enhanced ERK activity was observed to be related to activation of HER family receptors with more HER receptor dimerization and phosphorylation, increased in HER3 expression and binding of adaptor molecules of HER2 and HER3. There was reduction in cell proliferation and more cell death when PI3K inhibition was combined with either HER2 inhibition or MEK inhibition (which prevented the activation of ERK). That PI3K/AKT/mTOR and MAPK could be synergistic canonical pathways were identified by Kinome non-biased high throughput RNA interference screening (77). The anti-cancer efficacy was significantly greater than single agent when neratinib, a pan-HER2 TKI, was combined with mTOR inhibition (77%) or with MEK inhibition (77%).

Translational study with genomic profiling of HER2-amplified breast cancers showed that there was significantly more enrichment of mutations known to activate RAS-MAPK signaling in metastatic cancers than in primary cancers (78). The enrichment of PI3K/AKT pathway was also tested but it did not reach statistical significance. MAPK pathway activation was also found to promote resistance to HER2 kinase inhibitors leading to a shorter PFS. However, this switch to MAPK enrichment from loss of AKT dependence conferred MEK/ERK hypersensitivity with increase in effectiveness of MET inhibitor.

Intuitive proteins (pAKT and pERK) and genetic (PI3KCA) candidates were not found to be good biomarkers to differentiate PI3K/AKT from MAPK dependence (79). However, the combined measurement of three non-intuitive proteins comprising EGFR, HER3, and CDKN1B (a cyclin-dependent kinase inhibitor p27) on cell lines and primary tumor samples seemed more promising. From analysis of proteomic and cellular response data, functional relationship revealed that highly proliferative cells expressed increased level of EGFR and were MAPK pathway dependent. The slowly proliferating cells had higher levels of HER3 and CDKN1B and were PI3K dependent. The measurement of these three proteins helped to identify the mechanism of drug resistance, select the appropriate targeted drugs, and aid in design of new treatment strategies.

### Cyclin D1-CDK4/6-pRb pathway

Cyclin D1 and CDK4 were found to mediate therapeutic resistance to HER2 blockade in HER2-positive cancers (80). HER2 blockade caused a drop in cyclin D1 abruptly, but it rebounds to high level when tumor recurred. The growth seemed to be CDK4/6-dependent leading to phosphorylation of TSC2, mTORC1, and the downstream cascade. Moreover, the tumor recurrence and the cyclin D1 rebound were associated with hyperactivation of MAPK pathway. Further *in-vitro* experiments showed that stably expressing CCND1 (cyclin D1) in trastuzumab/lapatinib cell lines reduced the sensitivity to trastuzumab and lapatinib, indicating increased cyclin D1 level that confers resistance to HER2 pathway blockade. There was a similar observation in patients when the copy number of CCND1 was examined in a cohort of patients that received neoadjuvant chemotherapy and trastuzumab. The treatment failed to achieve pCR when there was a higher absolute CCND1 copy number.

### Resistance related to PD-L1 pathway and tumor microenvironment

HER2-positive cancer cells utilize the PD-1/PD-L1 axis to evade cytotoxicity by immune cells through suppression of CD8+ T cells and DCs. The induction of PD-L1 may be associated with changes in the tumor microenvironment. There may be more production of pro-angiogenic stimuli such as vascular endothelial growth factor (VEGF) and angiopoietin 2 (ANG2) that promote angiogenesis (81). The resultant abnormal tumor vasculature may contribute to hypoxia and reduction in pH in the tumor microenvironment leading to the production of PD-L1 by different immune and cancer cells, causing immunosuppression and reduction in adoptive immunity. However, classical anti-angiogenic therapy was not found to provide clinical benefit (82). Other vascular-normalizing treatment strategy should be designed to change the tumor microenvironment from an immunosuppressive to an immunosupportive one. One such strategy is to combine anti-angiogenic therapy with immunotherapy.

Other changes may include metabolism of tryptophan (Trp) in the tumor microenvironment generating crucial metabolites during its degradation. Tryptophan catabolism was found to play a key role as an important metabolic regulator of immunosuppression and cancer progression (83). The catabolism involves catalytic enzymes indoleamine-2.3-dioxygenase 1 (IDO1), IDO2, and trptophan-2,3-dioxygenase (TDO2). These enzymes deplete Trp and produce metabolites along the kynurenine pathway (KP), facilitating tumor immune evasion by induction of regulatory T (Tregs) cells and suppression of function of CD8+ and DC cells. IDO1 gene expression is high and KP is highly dysregulated in HER2 positive and triple negative breast cancers (84). The downstream KP enzymes are highly upregulated in these cancers leading to the production of potent immunosuppressive metabolites.

## Implications on the choice of therapy

As summarized in **Table 1**, the molecular and genetic analysis of the tumor samples are important to strategize treatment for the patients, especially in the neoadjuvant setting when the pathologic response can be assessed and further analysis is possible on residual cancer when the treatment is not successful. In this section, the clinical implication of those genetic and molecular factors on the choice of therapy for HER2-positive breast cancers are further discussed, with emphasis being placed on novel agents and combination therapies that are being investigated in clinical trials (**Tables 2**, **3**).

**Table 1:** Clinical implications of factors affecting metastasis and drug resistance in her2-positive breast cancers.

Factors	Clinical Implications
Mutations of HER2 receptor	Certain mutations like the L755S could result in resistance to trastuzumab and lapatinib. These HER2 mutations usually occur together with other gene mutations such as TP53 or PIK3CA. Treatment-resistant cells appear to retain some sensitivity to neratinib but may require combinational approach using MEK inhibitor and/or PI3K inhibitor.
HER2 GCN and HER2 heterogeneity	HER2 GCN is a biomarker of trastuzumab efficacy and prognosis. Biologic heterogeneity within HER2-positive BC could be a marker for pCR (and those with non-pCR may switch to another subtype).
Truncated HER2 receptor (p95)	Higher risk of failure and poor response to treatment with trastuzumab-based chemotherapy. Kinase activity is still retained, so those with p95 may still respond to TKI.
Gene mutational changes	TP53 and PIK3CA are the most common and could be related to cancer progression as well as affecting response to treatment. PI3K-inhibitors could play a role in treating HER2-positive BC, but warrants further investigations. The PIK3K/AKT pathway could be synergistic to the MAPK pathway, therefore MEK/ERK-inhibitors may prove to be useful in combination therapies.
Cyclin D1-CDK4/6-pRb Pathway	The CDK pathway could mediate resistance to HER2 pathway blockade. Combination therapies with CDK4/6-inhibitors and HER2-inhibitors for HER2-positive BC showed encouraging results and are continued to be investigated. Results from ongoing trials such as PATRICIA II, PATINA, and NA-PHER2 could provide important answers.
Resistance related to PD-L1 pathway and TME	The induction of PD-L1 may be associated with changes in the TME. By targeting an immunosuppressive TME and modifying it into an immunosupportive TME, the dual blockade of the HER2 and PD-1/PD-L1 treatment strategy could yield more benefit.

**Table 2:** Selected novel anti-her2 agents that are being developed.

Novel Anti-HER2 Agents	Pharmacology	Clinical Development Stage
Margetuximab	An anti-HER2 monoclonal antibody, with HER2 binding properties that are similar to trastuzumab, but with an engineered Fc region	Phase 3 in heavily pretreated HER2-positive metastatic breast cancer (85)
Ertumaxomab	A trifunctional BsAb targeting HER2 on tumor cells, CD3-expressing T cells, and Fc-gamma receptors on immune effector cells	Phase 2 in HER2-positive metastatic breast cancer that progressed after trastuzumab therapy (study terminated due to change in development plan) (86)
Trasintuzumab	A BsAb that consists of trastuzumab plus hersintuzumab, which is a novel anti-HER2 monoclonal antibody targeting a different epitope than trastuzumab and pertuzumab	Preclinical (cell lines and animal study) (87)
KN026	A BsAb that targets two separate epitopes of HER2 simultaneously	Phase 1 in pretreated HER2-positive metastatic breast cancer (88)
Zanidatamab	A BsAb that targets two separate epitopes of HER2 simultaneously	Phase 1b/2 in locally advanced/inoperable and/or metastatic HER2-positive breast cancer (89)
Trastuzumab deruxtecan (T-DXd)	An anti-HER2 ADC with a higher DAR as compared to T-DM1	Phase 3 in chemotherapy-pretreated HER2-low metastatic breast cancer (90)

**Table 3:** Selected ongoing clinical trials on combination therapies.

Clinical Trial	Combination Therapy Being Investigated	Expected Completion
EPIK-B2 (Phase III) (91)	Trastuzumab + pertuzumab + alpelisib in patients with PI3KCA-mutated, HER2-positive metastatic cancers that progressed after 4-6 cycles of taxane plus trastuzumab and pertuzumab	Mid 2025
(Phase II) (92)	T-DM1 + palbociclib in recurrent or metastatic HER2-positive breast cancer	Late 2024
PATRICIA II (Phase II) (93)	Trastuzumab + palbociclib + endocrine therapy in previously treated metastatic HER2-positive, HR-positive breast cancer	Mid-Late 2023
PATINA (Phase III) (94)	Trastuzumab ± pertuzumab + palbociclib + endocrine therapy in metastatic HER2-positive, HR-positive breast cancer	Mid 2023
TOUCH (IBCSG 55-17) (Phase II) (95)	Trastuzumab + pertuzumab + palbociclib + letrozole in postmenopausal HER2-positive, HR-positive breast cancer	Late 2022
APTneo (Phase III) (96)	Trastuzumab + pertuzumab + atezolizumab + chemotherapy in early high-risk and locally advanced HER2-positive breast cancer that is suitable for neoadjuvant therapy	Late 2026

### HER2 receptor inhibition

Under the usual clinical practice situation of HER2 overexpression, the standard approach of treatment is to target HER2 receptor with trastuzumab and pertuzumab. Various treatment combinations with and without chemotherapy have been tested successfully in the metastatic, neoadjuvant, and adjuvant situations. However, novel compounds are being developed and new treatment strategies are designed. One such new compound is margetuximab which is a monoclonal antibody. Though with similar specificity and affinity as trastuzumab, margetuximab may increase ADCC as it has four to five times higher affinity to bind to the activating Fc receptors of effector immune cells like NK cells and seven times lower affinity to bind to inhibitory Fc receptors of the antigen presenting cells (2). Its clinical activity was demonstrated in the SOPHIA trial when patients with cancers resistant to anti-HER2 therapies were treated with either margetuximab or trastuzumab in combination with chemotherapy (97). The PFS improved by 4.9 months with an ORR of 25.2% versus 13.7% (85).

Another new approach is to develop bispecific antibodies (BsAb). BsAb is a rapidly growing class of cancer therapeutics. It can simultaneously bind to different epitopes of the same antigen or different antigens altogether (biparatopic binding). It can interfere with multiple surface receptors and their associated ligands, inducing ADCC and complement-dependent cytotoxicity (CDC). Ertumaxomab is a trifunctional BsAb as it has two antigen recognition sites targeting HER2 and CD3-expressing T cells in addition to its isotype combination that binds to Fc-gamma receptors of immune effector cells. The compound brings the HER2 cancer cells to the mature T cells forming a T-cell/tumor cell aggregate to recruit cytotoxic immune cells such as NK cells. The recruited cells bind with their Fc-gamma receptors to aggregate and form a tri-cell complex leading to tumor cell lysis. A phase 1 study was performed which showed that ertumaxomab was safe with clinical activity (86). A phase 2 study was planned, but was terminated because of a change in development plan. Due to the potential benefit of BsAb, other compounds have been developed. Trasintuzumab, one such example, is composed of trastuzumab and hersintuzumab, a humanized anti-HER2 monoclonal antibody that recognizes a different epitope than trastuzumab and pertuzumab (87). Both variable domains are fully functional and induce tumor regression in nude mice bearing cancer xenograft.

The results of a phase 1 study that evaluated the first-in-human HER2-targeted BsAb KN026 for treating metastatic HER2-positive breast cancers were reported recently (88). KN026 binds to two distinct HER2 epitopes. KN026 monotherapy was administered in 63 patients who progressed on anti-HER2 therapies. The ORR was 28.1% and the median PFS was 6.8 months. The study also showed that co-amplification of CD12 could be a promising biomarker of response.

Zanidatamab (ZW25), like KN026, has high affinity of binding to two epitopes of HER2, the juxtamembrane (trastuzumab) domain (EDC4) and dimerization (pertuzumab) domain (EDC2), causing receptor clustering, downregulation of HER2, inhibition of signal transduction, and enhancement of ADCC. The clinical data were presented at the San Antonio Breast Cancer Symposium 2021. Zanidatamab monotherapy decreased targeted lesions in the majority of patients with HER2-positive breast cancers (98). A phase 1 trial showed that zanidatamab in combination with chemotherapy was well tolerated and demonstrated encouraging anti-tumor activity in heavily pretreated patients with a confirmed ORR of 36.4% and median PFS of 7.2 months (99). Another ongoing phase 1b/2 study on zanidatamab in combination with docetaxel, but as the first-line treatment of advanced HER2-positive breast cancer patients, has also shown an encouraging result with a confirmed ORR of 90.5% and a 6-month PFS rate of 95.2% (89).

The situation with HER2-low may be different. Though trastuzumab and pertuzumab might be less effective in cancers with HER2-low, antibody-drug conjugate (ADC) such as trastuzumab emtansine (T-DM1) and trastuzumab deruxtecan (T-Dxd) could still be effective as long as the agent could bind with the HER2 receptor releasing emastine or deruxtecan to cause cell kill. T-DXd has been shown to be significantly effective over physician’s choice of chemotherapy (capecitabine, eribulin, gemcitabine paclitaxel, or nab-paclitaxel) in improving PFS of metastatic patients with HER2 score of 1+ or 2+ on immunohistochemistry or with negative *in-situ* hybridization score (90).

Both T-DM1 and T-Dxd use IgG1 as the monoclonal antibody to target the HER2 receptor. IgG1 can induce a stronger ADCC and CDC. However, that T-Dxd could be more effective than T-DM1 could be related to a higher drug to conjugate ratio (DAR) (100). The DAR of T-DM1 is 3.5 whereas of T-Dxd is 8. Moreover, the payload of the former is DM1, which is a tubulin polymerization inhibitor, whereas that of the later is a topoisomerase 1 inhibitor. Both have a higher IC50 than conventional chemotherapy. However, the linker of T-Dxd is cleavable whereas that of T-DM1 is non-cleavable. This property of T-Dxd together with a highly membrane permeable payload enhances the bystander effect (over T-DM1) to kill neighboring HER2-negative cancer cells in the tumor microenvironment. Phase 1 study has shown that T-Dxd had activity in patients previously treated with T-DM1 (101). The phase 2 study (Destiny 01) confirmed that T-Dxd was effective in patients resistant/refractory to T-DM1 with an ORR of 60.9% (6% achieved complete response), clinical benefit ratio (CBR) at 6 months of 76.1%, and median duration of response (DOR) of 14.8 months (102).

However, in resistant cancers, the HER2 receptor may be truncated and thus the testing of p95 is important. Those cancers with truncated HER2 receptors would not respond to monoclonal antibody-based therapies, which require the binding with the HER2 receptor to be effective (103). Thus, these patients should not be treated with trastuzumab, pertuzumab, or any of the ADC like T-DM1 or T-DXd. Similarly, when the HER2 receptors are mutated with L755S and K735E mutations or when there is HER2 heterogeneity, the use of these antibodies would not be so effective.

Under these situations (including some patients with HER2-low), the treatment strategy should include one of the TKIs (lapatinib, neratinib, pyrotinib, and tucatinib). Lapatinib and neratinib were found to be effective in cancers with p95 expression (104, 105). HER2 mutations might be enriched more in invasive lobular cancers and they might be the result from treatment selective pressure, conferring resistance to trastuzumab. However, such cancers that carried the mutations might still be sensitive to neratinib even when they lost HER2 amplification (106, 107).

### Signal transduction pathways inhibition

PIK3CA mutation is usually associated with aggressive cancers that do not respond well to neoadjuvant therapy. Identification of its presence would be useful to design novel treatment. Compared to single agent neratinib, combining it with everolimus (an mTOR inhibitor) significantly inhibited cancer growth with marked suppression of Ki67 and enhancement of apoptosis (77). In patients found to have trastuzumab resistance, the combination of everolimus with trastuzumab and chemotherapy improved survival (108). The benefit of the triple therapies was mostly found in cancers that lost PTEN.

Alpelisib (a PI3K-alpha specific inhibitor) was combined with T-DM1 in a phase 1 study on metastatic HER2-positive breast cancer patients who were resistant to trastuzumab-based therapy (109). The results were impressive with an ORR of 43% and CBR of 71%. The median PFS was 8.1 months. A phase III multicenter study (NCT04208178) is undergoing to evaluate the combination of alpelisib with trastuzumab and pertuzumab in patients with PI3KCA-mutated metastatic cancers that progressed after 4-6 cycles of taxane plus trastuzumab and pertuzumab (91).

The activation of MAPK pathway is a relatively late event and this may include the activation of the cyclin D1-CDK4/6 axis. These cancers may eventually require more aggressive treatment with chemotherapy. To differentiate whether it is the PI3K or MAPK pathway that predominates, checking of EGFR, HER3 and CDKN1B may help and treatment can be strategized differently. If MAPK pathway eventually interacts with the cyclin D1-CDK4/6 axis, the detection of pRb is helpful when CDK inhibitors can be used to treat the patients.

NF-kappaB pathway is related to cancer cell invasiveness and inhibition of its activation could bring more TILs into the tumor and thus improved response to trastuzumab and chemotherapy. Anti-inflammatory drugs such as aspirin and sodium salicylate are shown to degrade IkappaBalpha and inhibit key components of the canonical pathway of NF-kappaB (110). In addition, inhibition of TNF-alpha, an activator and effector of NF-kappaB, was found to have partial disease stabilization effects. However, whether by anti-inflammatory drugs or anti-TNF-alpha antibodies like infliximab, the blockage might not be sufficient to control cancer invasion and combining it with conventional chemotherapy and targeted therapies might improve the treatment results.

### CDK and HER2 inhibition

Cyclin D1-CDK4/6 axis could play a key role in trastuzumab resistance. Experiments on CDK4/6 inhibition showed that suppression of Rb phosphorylation would not only induce a partial G1 arrest, but also reduction of TSC2 phosphorylation and partial suppression of mTORC1 and S6K activities (80). As a result of the suppression of S6K, the feedback inhibition of EGFR family kinases would be relieved and AKT phosphorylation would be increased, making the cells re-sensitize to anti-HER2 treatment. Although there was suppression of cell proliferation, the effects were mild. However, when there was concurrent inhibition of both the CDK and HER2 pathways, synergy was observed and there was maximal suppression of TSC2 phosphorylation and complete shutdown of S6K. Because of the HER2 inhibition, the relieved feedback inhibition of the receptor kinases would not activate AKT. Thus, reduction of cell proliferation was more pronounced.

Clinical trial testing the combination of palbociclib and trastuzumab in HER2-positive metastatic breast cancer (PATRICIA) showed superior results for luminal cancers than non-luminal ones (111). The CBR was significantly higher (73% vs. 31% respectively), but the PFS did not reach statistical significance though there was a difference in median PFS of 8 months (12.4 months vs. 4.1 months respectively). A phase 1b/2 study on ribociclib plus trastuzumab or T-DM1 in patients with advanced HER2-positive breast cancer previously treated with trastuzumab, pertuzumab, and T-DM1 was conducted (112). Analysis on the ribociclib and trastuzumab combination showed that the treatment was safe with one patient experiencing a stable disease for more than 24 weeks. However, there was no objective response observed and there was no meaningful analysis of PFS. This study also included nine evaluable patients treated with T-DM1 plus ribociclib (113). The treatment was well tolerated, the ORR was 33%, and the median PFS was 12.5 months. Another similar phase 1/1b study was performed to evaluate the combination of T-DM1 with palbociclib after trastuzumab and taxane (114). The ORR was 33%, the median PFS was 6 months, and the median DOR of responders was not reached. A phase 2 study on this palbociclib and T-DM1 combination is being tested in a phase 2 trial and it is expected to be completed in December 2024 (NCT03530696) (92).

For triple positive cancers, the combination of anti-HER2 treatment, CDK inhibitor, and hormonal treatment are being evaluated. The monarcHER study is a randomized phase 2 clinical trial to evaluate another CDK inihibitor, abemaciclib, for hormone receptor-positive and HER2-positive advanced breast cancer (115). The patients were randomized to receive either abemaciclib plus trastuzumab with or without fulvestrant versus trastuzumab plus standard of care chemotherapy. The median PFS was significantly better when patients received abemaciclib, trastuzumab, and fulvestrant than trastuzumab plus standard of care chemotherapy (8.3 months vs. 5.7 months). Besides superior results, the chemotherapy-free regimen showed a tolerable safety profile. The PATRICIA II phase 2 study then evaluates palbociclib plus trastuzumab and endocrine therapy versus treatment of physician’s choice in metastatic HER2-positive, hormone receptor-positive breast cancers (93). Tumor and blood samples are collected for biomarker analysis. Clinical study with similar design to evaluate the value of palbociclib for the same kind of patients (PATINA) is also conducted (94). Both PATRICIA II and PATINA trials have not been completed and the trial results are expected to be released next year.

Chemotherapy-free regimen was also tested in the neoadjuvant setting. The NA-PHER2 study was an exploratory open-labeled phase 2 neoadjuvant study on the treatment with trastuzumab and pertuzumab plus palbociclib and fulvestrant (116). Of the 30 evaluable patients, the clinical ORR was 97% immediately before surgery. Eight patients (27%) achieved pCR in both breast and axilla. The geometric mean Ki67 dropped from 31.9 at baseline to 4.3 at week 2, but it increased again to 12.1 at surgery. The geometric mean of apoptosis was 1.2 at baseline, but it dropped to 0.4 at surgery. The TOUCH (IBCSG 55-17) trial is a phase 2 open-label neoadjuvant study the combination of trastuzumab and pertuzumab with either palbociclib and letrozole or weekly treatment with paclitaxel for elderly patients (95). The trial is expected to be completed in August 2022. More trials are expected to be conducted to assess whether triple targeting without chemotherapy could be a safe but effective treatment strategy.

### Immune checkpoint and HER2 inhibition

PD-L1 is frequently expressed in HER2-positive cancers and the dual blockage of HER2 and PD-L1 pathways could be an attractive strategy. Multiple clinical trials have been conducted and these involve mainly pembrolizumab, atezolizumab, and durvalumab on metastatic breast cancers. However, other immune checkpoint inhibitors are also being evaluated, such as avelumab being evaluated in the AVIATOR trial and nivolumab being evaluated in another phase 1b trial. The APTneo trial is a phase 3 neoadjuvant study to evaluate atezolizumab, pertuzumab, and trastuzumab with chemotherapy (versus trastuzumab, pertuzumab, and chemotherapy) in early high-risk and locally advanced HER2-positive cancers (NCT03595592) (96). It is planned to recruit 650 patients to assess whether the experimental treatment could improve event-free survival (EFS) and secondarily pCR, distant EFS, and overall survival.

The PANACEA trial was a single arm phase 1b/2 clinical study to evaluate the combination of pembrolizumab and trastuzumab in trastuzumab-resistant HER2-positive cancers (117). The phase 2 part included 52 patients and the benefit was mainly found in PD-L1-positive patients with an ORR of 15% versus 0% in PD-L1-negative patients. However, the median PFS was not significantly different between the two groups.

The KATE2 trial was another clinical study to evaluate immunotherapy in HER2-positive breast cancers (118). The study was a phase 2 double-blind randomized controlled trial in a 2:1 assignment that included 330 patients who received either T-DM1 alone or in combination with atezolizumab. There was no difference in median PFS for the two groups (8.2 months vs. 6.8 months). Moreover, serious adverse events occurred more often in the study group (33% vs. 19%). Similar to the results of the PANACEA trial, the benefit seemed to occur mainly in PD-L1 positive cancers.

Durvalumab is another monoclonal antibody against PD-L1. It was tested as a combination treatment with trastuzumab in a phase 1b study on HER2-positive metastatic breast cancers, previously treated by chemotherapy and anti-HER2 treatment (119). All of the 15 recruited patients were tested negative for PD-L1 and no significant clinical activity was observed.

BsAb have been developed to target both HER2 and PD-L1 (120, 121). These will direct T cells to engage HER2 cells blocking simultaneously both HER2 downstream signals and PD-1/PD-L1 axis to increase ADCC. One particular BsAb was constructed by fusing anti-PD1 scFv with the effector-functional Fc part of the IgG (trastuzumab) *via* a flexible peptide linker (120). There was high affinity binding with HER2 and PD-1 with potent anti-tumor activities. The crosslink of HER2-positive cells with T cells to form a PD-1 immunological synapse direct T cell cytotoxicity without the need of antigen presentation. The BsAb can also potentially activate T cells in TME *via* PD-1/PD-L1 blockade. However, this BsAb adopts an unmodified IgG1 as the backbone and this may limit its clinical effectiveness. Besides ADCC, there are other effector functions that include CDC and antibody-dependent cellular phagocytosis (ADCP), which can lead to collateral damage to PD-1-expressing tumor-infiltrating T cells. The other BsAb reported was constructed with the whole trastuzumab IgG and tandem anti-PD-L1 scFVs in the format of IgG-scFV (121). The BsAb generated was more stable with more enhanced ADCC than trastuzumab, especially in the late phase of the disease model using PMBC humanized tumor xenograft.

Research on other novel treatment strategies are being conducted and anti-cancer vaccines are actively being studied. The anti-HER2 vaccines include peptide-based and other epitope-based vaccines, gene-based vaccine, and whole cell vaccine (122). Spontaneous antibody production by the peptide- or epitope-based vaccines is uncommon and therefore require combination with granulocyte-macrophage colony stimulating factor (GM-CSF). Different domains of HER2 peptide were evaluated as antigens. These peptides are located at the ECD (E75), transmembrane portion (GP2), and intracellular domain (AE37). Another formulation of this class of vaccine includes a mixture of peptide sequences derived from the ECD and ICD – the anti-recombinant HER2 protein (dHER2) vaccine.

The gene-based vaccines include the viral vector-based vaccine and the plasmid vector-based vaccine. Both formulations deliver DNA sequences coding for HER2 to induce antibodies against both viral and non-viral antigen and cell mediated immune responses *via* increase in CD8+ T cell response. The whole cell vaccines include tumor cell-based vaccine which has poor immunogenicity and requires GM-CSF, dendritic cell-based vaccine which induces significant cell mediated anti-HER2 immune response, and autologous T cell-based vaccine which is an adoptive transfer of autologous HER2 specific T lymphocytes. Another form of adoptive T cell transfer is chimeric antigen receptor (CAR)-T cell therapy which uses patient’s own immune cells to combat against cancer. It has shown great benefit in hematological malignancies but there are more studies on solid cancers including HER2 positive breast cancers (123). HER2-specific CAR-T cells can eradicate trastuzumab-resistant breast cancer xenograft and can penetrate the tumor matrix which is a barrier of most monoclonal antibodies (124).

## Conclusion and future direction

The translational research on the genetic and molecular mechanisms of cancer progression and treatment resistance provides an insight into the planning of treatment strategies for HER2-positive breast cancers. The HER2 pathway is unique as its dimerization is linked to multiple signal transduction pathways and importantly, the crosslink between these pathways continues to be evaluated in basic research and clinical trials. An important, but yet to be elucidated connection is between the HER2 pathway and the immune checkpoint pathway; PD-L1 gene can be activated to produce PD-L1 to inhibit the TILs thereby impairing their ability to kill cancer cells and also reducing the effectiveness of anti-HER2 monoclonal antibodies including pertuzumab, ADC, and BsAB.

The treatment landscape for HER2-positive breast cancers within the next decade, especially for treatment-resistant, metastatic cases, will likely be shaped by the results of clinical trials investigating the use of novel ADCs and BsAb. Standard treatment using a single monoclonal antibody against HER2, or a combination of two monoclonal antibodies for node-positive cases, will likely continue to be used for patients with the usual HER2 overexpression. In some selected patients when the hormonal receptors are negative and the cancer load is low, chemotherapy-free regimen can be considered.

However, in patients with HER2 mutation or when the HER2 receptor expression is low, the standard treatment with trastuzumab and pertuzumab may not give the desired treatment response. These patients may be better treated with ADC such as T-DM1 or T-Dxd. TKI can be another consideration, especially in patients with truncated HER2 receptors (p95), but ADC may have a greater treatment impact than TKI when there is PI3K mutation.

For cancers which expressed both hormonal receptors and HER2, the cyclin D1-CDK4/6 axis may be involved in the cancer cell progression. The treatment option can include inhibition of both pathways with hormone therapy, CDK inhibitor, and anti-HER2 treatment while doing away with chemotherapy. For cancers that also expressed PD-L1, the dual targeting of HER2 and PD-L1 pathways may improve the treatment outcome, especially in late advanced cancers.

A number of clinical trials evaluating the concepts listed are being conducted. As we continue to improve our understanding of the biology of HER2-associated pathways, it remains to be elucidated whether the design of these novel agents and treatment strategies will eventually benefit the patients in the clinic.

## Author contributions

CC and LC wrote the first draft of the manuscript and drew the figures. EL and C-HW revised and edited the manuscript. All authors contributed to the article and approved the submitted version.

## Conflict of interest

The authors declare that the research was conducted in the absence of any commercial or financial relationships that could be construed as a potential conflict of interest.

## Publisher’s note

All claims expressed in this article are solely those of the authors and do not necessarily represent those of their affiliated organizations, or those of the publisher, the editors and the reviewers. Any product that may be evaluated in this article, or claim that may be made by its manufacturer, is not guaranteed or endorsed by the publisher.
